# Long-lasting IgM and declining IgG levels: a serologic 5-year follow-up study in healthy blood donors infected with hepatitis E virus

**DOI:** 10.1007/s00430-025-00838-y

**Published:** 2025-06-04

**Authors:** Ricarda Plümers, Jens Dreier, Cornelius Knabbe, Tanja Vollmer

**Affiliations:** Herz- und Diabeteszentrum Nordrhein-Westfalen, Universitätsklinik der Ruhr-Universität Bochum, Medizinische Fakultät OWL (Universität Bielefeld), Georgstr. 11, Bad Oeynhausen, Germany

**Keywords:** Hepatitis E virus, Serology, Blood donors, Asymptomatic infection

## Abstract

Hepatitis E virus (HEV) has attracted increasing attention in transfusion medicine in recent years. Mandatory testing regimes in Europe have resulted in not only ensuring the safety of blood products, but also providing information on the spread and immunology of HEV infections. We tracked a cohort of 497 donors identified as HEV RNA-positive during blood donation. Several follow-up samples were collected and serologically analyzed for 370 of them, up to five years after the index donation. In addition to the expected increase in immunoglobulins M (IgM) and G (IgG) titers at the beginning and the decrease over the years, we observed a proportion of 7.3% with positive anti-HEV IgM (long-term IgM-positive) and 9.1% with negative anti-HEV IgG (seroreversion) in five-year follow-ups, determined by serological tests from three different manufacturers. Both phenomena have an impact on the assessment of the correlation between incidence and seroprevalence. They are dependent on the sensitivity and specificity of serologic assays used and have a sex bias, which indicates a stronger, longer-lasting humoral immune response in women. These data offer new insights into the long-term development of immunity to HEV and thus complement short-term epidemiological data on the incidence and seroprevalence that have been obtained so far.

## Introduction

In 1983, Balayan et al. first described a virus that was named hepatitis E virus (HEV) in 1990 and later classified as the species *Paslahepevirus balayani* [1, 2]. Initially thought to be transmitted between humans via the water-borne route, occurring particularly in flooded areas in Africa and Asia, HEV was also detected in pigs in 1997 [3, 4]. As it turned out, HEV, specifically genotypes 3 and 4, is transmitted via the food-borne route, especially through undercooked meat, with genotype 3 being widespread in Europe [5]. However, most infections are asymptomatic, impeding the recording of exact epidemiologic numbers, although HEV is a notifiable infection in Germany. The latter is, not least, because infection can lead to severe complications in immunocompromised patients [6]. In addition to infection via the food-borne route, these patients may get infected by receiving an HEV RNA-positive blood transfusion, which is why the testing of blood donations is mandatory in various European countries, including Germany [7].

The wide spread of HEV in Europe was highlighted by serological studies. The early phase of convalescence is determined by positive IgM titers (maximum reached after 36 days) while IgG can contribute to long-term immunity (maximum reached after 52 days) [8]. Anti-HEV IgG seroprevalences of up to 52.5% (in South West France) in Europe came to light [9, 10]. The detection of anti-HEV antibodies in blood donors revealed seroprevalences of up to 29.5% in Germany [11–13]. The variance of this parameter in different studies, even within a single country, arises from regional influences (e.g. local specialties from raw meat), and from different sensitivities of the serological assays used [9, 14].

The increasing data from mandatory blood donor screening has provided valuable insights into both seroprevalence and the actual number of infections [15–19]. In addition, donors have been allowed to donate again after a provision period opening the possibility of tracking the serostatus over years [20].

We have been carrying out standard HEV screening in donors since 2015. From 2015 to 2022, a cohort of 497 HEV positive index donors were identified whose epidemiological data have been published recently [21]. Here, we present the data from serological analysis of the follow-up donations up to five years after the index donation in this cohort.

## Methods

### Blood donors and HEV nucleic acid amplification test (NAT) screening

Between 2015 and 2022, 497 HEV-RNA positive out of 731,630 allogenic blood donations were identified at Uni.Blutspendedienst OWL, located in North Rhine-Westphalia, Lower Saxony and Hesse. The proportion of females was 41.5% in the whole cohort and 26.5% among HEV RNA-positive donors. Further epidemiological data and the NAT strategy are published elsewere [21].

In brief, automated total RNA extraction was performed on the NucliSens easyMAG (bioMerieux, Nürtingen, Germany), AltoStar AM16 (Altona Diagnostic Technologies, Hamburg, Germany) or the cobas 6000 (Roche Diagnostics, Basel, Switzerland) systems in minipools of 96 samples. Real-time polymerase chain reaction was conducted using the RealStar HEV RT-PCR Kit (ADT), AltoStar HEV RNA RT-PCR Kit (ADT) and the cobas HEV assay (Roche Diagnostics, Basel, Switzerland) on the Bio-Rad CFX96 system (Bio-Rad, Hercules, CA, USA) or on the cobas 6000 system (Roche Diagnostics).

All donors fulfilled the guidelines for administration to donation and claimed no known risk of viral infections. Donors were able to be readministrated for donation after being checked for HEV-RNA negativity four weeks past the index donation or after four months by mandatory testing. Routine follow-up testing for HEV RNA-negativity was conducted.

### Serologic testing

Readministration samples were collected from donors who returned to donate, allowing for the confirmation of seroconversion. Follow-up samples were sorted into samples of approximately three, six, nine months, one, two, three and five years after the index donation provided that the donors turned up to donate during those periods. Follow-up samples were collected until December 2023.

The serologic status was determined by the measuring HEV-specific IgM and IgG using the anti-HEV ELISA Kits from Euroimmun Medizinische Labordiagnostika AG (Lübeck, Germany; hereinafter referred to as Euroimmun assay; positive above a signal to cut-off ratio [S/CO] above 0.8 (IgM) and above 0.8 RU/ml (IgG); sensitivity: 100% (IgM), > 95.5% (IgG); specificity: > 97.8% (IgM), > 97.8% (IgG)). For samples exceeding the assay's quantification limit (> 25 RU/mL), dilution was applied. Additionally, the serostatus was confirmed using the Wantai anti-HEV ELISA Kits (Sanbio, Uden, Netherlands; hereinafter referred to as Wantai assay; positive for S/CO > 1; sensitivity: > 96.4% (IgM), 100% (IgG); specificity: > 95.3% (IgM), > 99.1% (IgG)) or the recomLine anti-HEV ELISA Kits (Mikrogen, Neuried, Germany; hereinafter referred to as Mikrogen assay; positive above 20 U/ml; sensitivity: 98.9% (IgM), 98.9% (IgG); specificity: 98.6% (IgM), 98.5% (IgG)) in case of an unexpected result, such as long-lasting IgM (six months or more) or disappearing IgG after detection in a prior donation. The choice of test system is based on previous study and automated sample processing in our laboratory at the beginning of our study ten years ago and avoid a bias resulting by changing the assay [22].

### Statistical analysis

Graphical presentation and statistical analyses were carried out with GraphPad Prism 9.0 software (GraphPad Software, San Diego, CA, USA). Statistical significance defined as *p* < 0.05 was calculated with the nonparametric two-tailed Mann–Whitney U tests.

## Results

A total of 497 donors with an initial HEV-RNA positive donation (index donation) were tracked, 370 of them presented themselves for readministration as donors. Blood samples for all of them were HEV RNA-negative within 140 d in mean (range 29–2640 d) after the initial HEV RNA-positive donation (readministration donation). Reinfection proven by HEV RNA-detection has not been observed. 357 donors showed reactive results for anti-HEV IgM and/or IgG in the index donation or the first follow-up donation. in the Euroimmun assays. Antibodies in the remaining 13 samples were detectable with the other two serologic assays (Wantai and Mikrogen assays). 28 donors had an IgM−/IgG+ serostatus at their index donation. 20 of them never had an IgM titer above the cut-off. We observed an IgM−/IgG+ serostatus at the index donation and conversion to IgM+/IgG+ in follow-up samples in eight cases when analyzing with the Euroimmun assay confirming it in three of them with one of the other two assays.

Follow-up samples were collected in intervals of around three, six, nine months, one, two, three and five years. The number of samples collected for the respective time periods, the time spans and the distribution of male to female donors represented by the proportion of female donors are shown in Table 1.

**Table 1:** Main characteristics of the cohort for every follow-up period including the number of samples available, the time point of sampling relative to the index donation and the proportion of female in the subgroup under consideration

Sample group	Number of samples	Mean time point of sampling (min/max) [days]	Proportion female [%]
Index donation	497	na	25.9
Readministration donation	370	140 (29/2640)	24.2
3 months	172	106.5 (29/135)	22.4
6 months	196	185 (136/224)	23.0
9 months	196	272 (225/315)	23.0
1 year	271	383 (316/571)	23.0
2 years	232	733 (581/912)	21.3
3 years	193	1109 (925/1500)	24.2
5 years	110	1846 (1500/2708)	22.8

na = not applicable

Samples were classified according to the serostatus via anti-HEV IgM and IgG results. Based on the time course of an infection, a change from IgM−/IgG− to IgM+/Ig− and IgM+/IgG+ to IgM−/IgG+ is expected. In case of unexpected results defined as an IgM+ titer six months and longer or a disappearing IgG− titer after more than six months with the Euroimmun assay, the samples were further analyzed with two ELISA kits from other manufacturers. Wheater the results were positive or negative was decided based on at least two matching test results (2:1 rule) (Table 2).

**Table 2:** Proportions of samples according to the time after the index donation divided by serostatus

Sample group	Shares of serostatus in the sample group [%]*				Total [%]*		Confirmed results 2:1 rule [%]**	
	IgM−/IgG−	IgM+/IgG−	IgM+/IgG+	IgM−/IgG+	IgM+	IgG−	IgM+	IgG−
Index donation	78.4	1.2	11.9	8.5	13.1	79.6		
Readministration donation	5.1	0.3	43.4	51.2	43.7	5.4		
3 months	1.2	0.6	55.4	42.9	56.0	1.8		
6 months	1.0	0.5	40	58.5	40.5	1.5	36.7	0.5
9 months	5.6	1.5	26.7	66.2	28.2	7.1	27.4	1.0
1 year	7.7	1.1	25.1	66.1	26.2	8.9	25.1	1.1
2 years	13.8	0.4	16.8	69.0	17.2	14.2	16.3	3.4
3 years	19.4	1.0	12.0	67.5	13.0	20.4	12.4	3.6
5 years	36.4	0.0	9.1	54.5	9.1	36.4	7.3	9.1

^*^Results obtained using the anti-HEV ELISA Kit from the manufacturer Euroimmun

^**^In case of an unexpected serology (IgM+ six months and longer, no IgG at readministration or IgG− after more than six months) the result was checked with a second anti-HEV ELISA Kit results obtained using the Mikrogen or Wantai ELISA. Results were determined based on two matching results (2:1 rule)

Overall, 56.0% (three months), 40.5% (six months), 28.2% (nine months), 26.2% (one year), 17.2% (two years), 13.0% (three years) and 9.1% (five years) of samples were detected to be anti-HEV IgM-positive with the Euroimmun assay. Results of long-term anti-HEV IgM positivity were confirmed in 36.7% (six months), 27.4% (nine months), 25.1% (one year), 16.3% (2 years), 12.4% (three years), 7.3% (five years) with a second test. 2.9% (8 of 280) of all donations classified as IgM-positive with the Euroimmun ELISA kit were disproved with the other two assays. It should be noted that the result of the ELISA from Wantai was positive in only 51.6% of all Euroimmun assay long-term IgM positive samples detected over the years, while that was the case for 94.7% regarding the Mikrogen assay.

Furthermore, it was observed that 1.8% (three months), 1.5% (six months), 7.1% (nine months), 8.9% (one year), 14.2% (two years), 20.4% (three years) and 36.4% (five years) of the donors to be anti-HEV IgG-negative with the Euroimmun assay. Seroreversion was confirmed for 0.5% (six months), 1.0% (nine months) 1.1% (one year), 3.4% (two years), 3.6% (three years) and 9.1% (five years) of all donors. It should be noted that in 17.2% of cases the Wantai assay confirmed a negative anti-HEV IgG result and in 10.4% of cases the assay of the Mikrogen assay did.

**Fig. 1:**
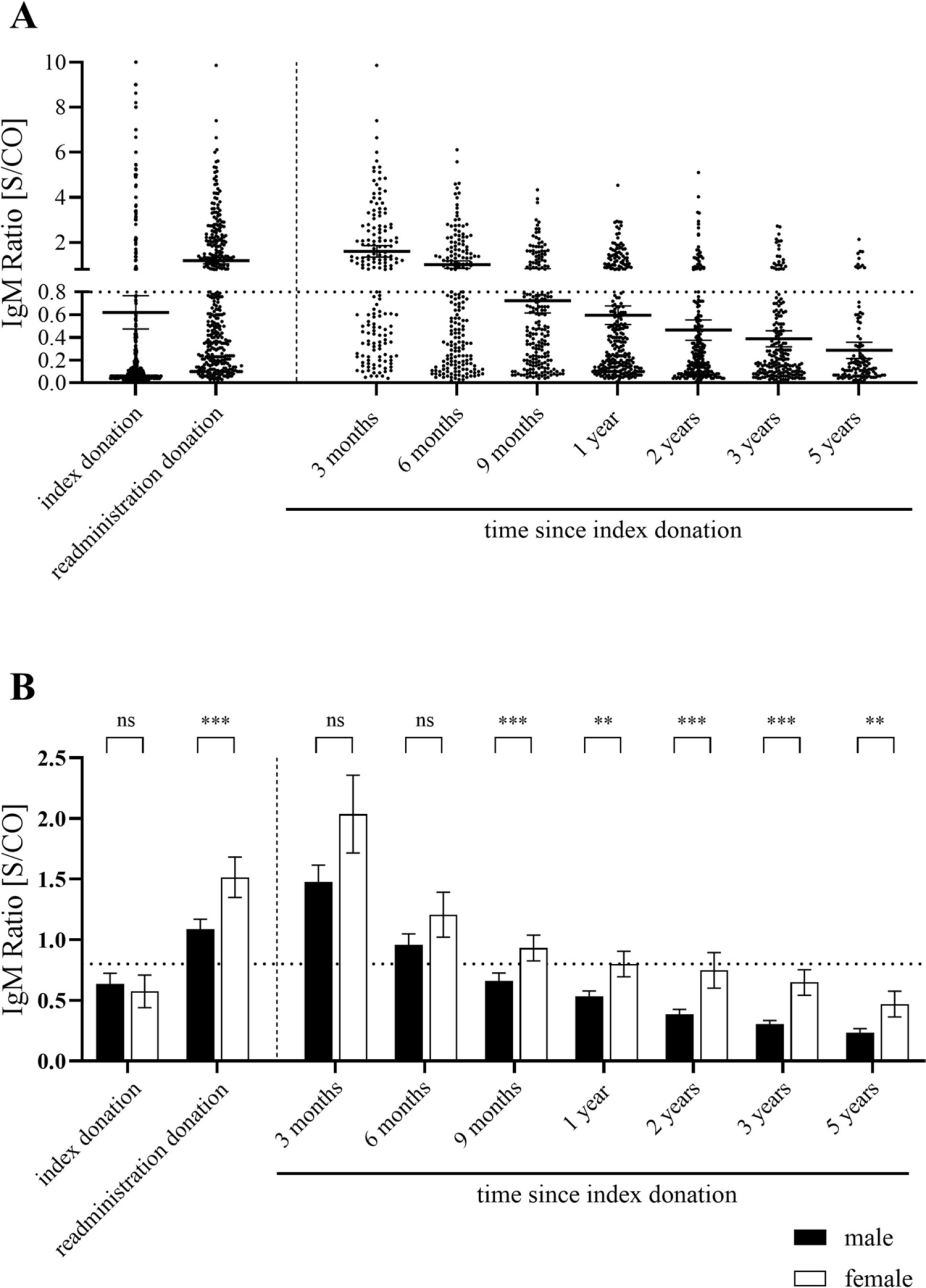
**A** S/CO of anti-HEV IgM measured via the Euroimmun assay. **B** Comparison of mean S/CO plus standard error of the mean in male and female donors. Dashed horizontal lines represent threshold for positive results. Significance levels according to the Mann–Whitney-U test: ns (not significant, *p* > 0.05), ** (0.001 < *p* < 0.01), *** (0.0001 < *p* < 0.001)

**Fig. 2:**
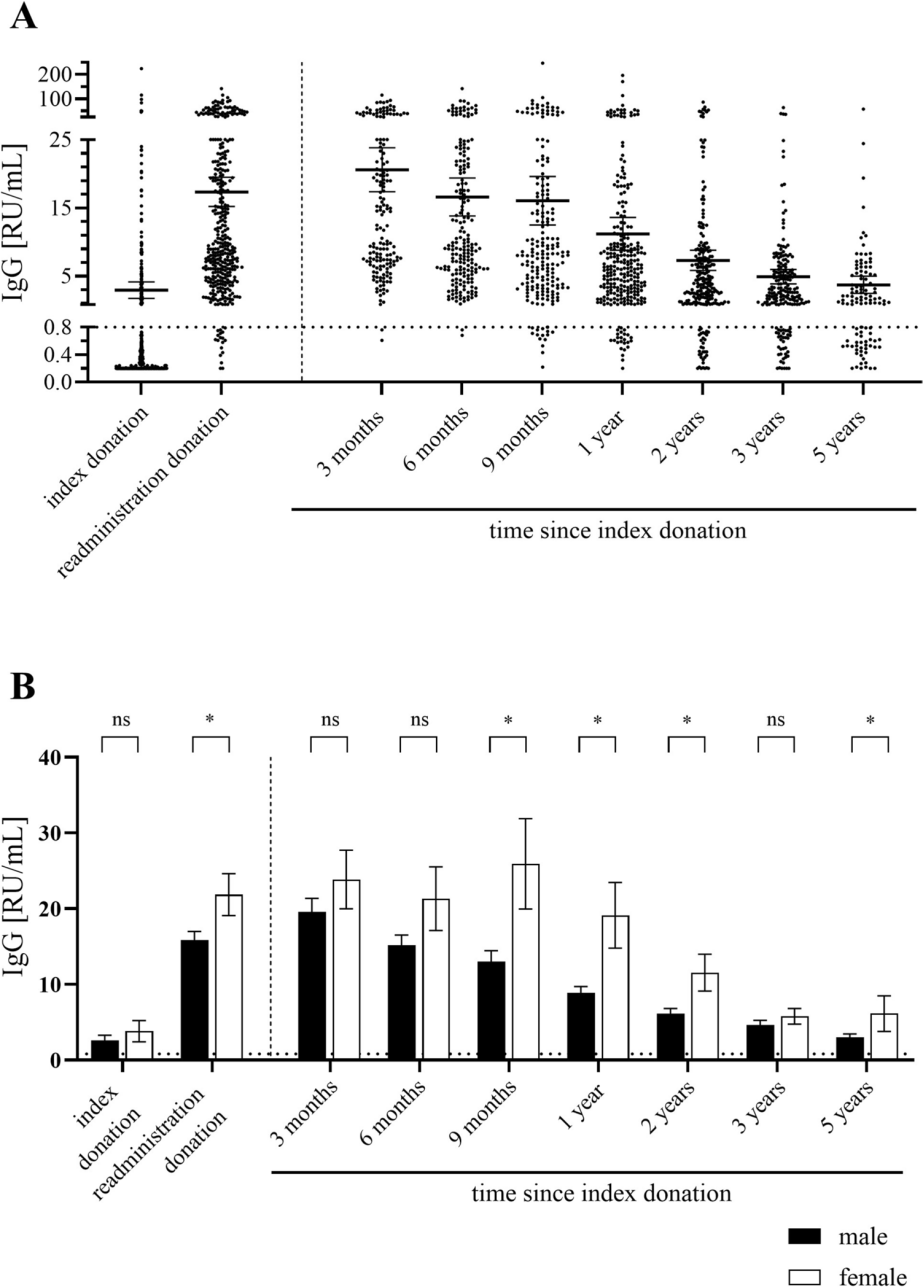
**A** RU/ml of anti-HEV IgG measured via the Euroimmun assay. **B** Comparison of mean RU/mL plus standard error of the mean in male and female donors. Dashed horizontal lines represent threshold for positive results. Significance levels according to the Mann–Whitney-U test: ns (not significant, *p* > 0.05), * (0.001 < *p* < 0.05)

In addition to the qualitative classification regarding the detection of anti-HEV IgM and IgG, a semi-quantitative S/CO ratio for IgM and a quantitative result in relative units (RU)/mL for IgG can be specified with the Euroimmun assay. These values were determined for each sample and plotted for the various sample groups according to the time after the index sample separated for IgM and IgG and men and women (Figs. 1 and 2).

The expected course for the detection of IgM is reflected in the S/CO determined with the Euroimmun ELISA. An increase in the average values is observed when comparing the index donation to the readministration donations. Moreover, the average S/CO in donations made three or six months after the index donation exceed the cut-off for the definition of an IgM-positive status. The average S/CO falls under the cut-off nine months following the index donation (Fig. 1A).

We found a different proportion of female donors in the long-term IgM-positive donors compared to the overall cohort (see Table 1, between 21.3 and 25.9%). Among the total number of long-term anti-HEV IgM-positive donors confirmed at one or more time points, 41 were female and 79 were male (proportion of female donors 34.2%). This observation is accompanied by significantly different S/CO values in men and women, especially in the long-term course after HEV infection (more than nine months) (Fig. 1B).

The IgG titers in the index donation is, on average, significantly lower than in the readministration donation, since most donors are anti-HEV IgG-negative. Furthermore, the IgG titer is highest in the early phase of convalescence (three months), but then decreases (Fig. 2A). The values remain, on average, above the cut-off limit of the manufacturer (> 0.8 RU/ml), but, especially after five years, isolated donations are below this limit and are therefore considered anti-HEV IgG negative.

Although the classification into IgG-negative and positive individuals leads to discrepancies between the assays, the quantitative values for anti-HEV IgG in those negative with the Euroimmun assay is closer to the cut-off than in the total group for the two comparison assays underlining the low-titer sensitivity phenomenon (example after one year; Mikrogen: 47.15 U/mL in the Euroimmun IgG negative, 100.02 U/mL in the total cohort; Wantai: 4.55 S/CO in the Euroimmun IgG negative, 10.67 S/CO in the total cohort).

Similar to anti-HEV IgM, differences were also found between men and women regarding anti-HEV IgG titers. Among the total number of anti-HEV IgG-negative donors confirmed at one or more time points, 11 were men and 2 were women (proportion of females 15.3%). Meanwhile, the female proportion was 30.1% among all IgG positive donors five years after their index donation. Accordingly, the titer values differed significantly between the two sexes, particularly in the late phase of recovery (nine months, one year, two years, five years).

## Discussion

Serological tests are a suitable tool for describing the epidemiology of an infection independently of the acute infection represented by NAT. HEV gained attention, due to the observed high seroprevalence long before studies on acute infections via NAT, for example among blood donors, were published. Nevertheless, little is known about the long-term titers of immunoglobulins against HEV although these are necessary to assess a correlation between incidence and seroprevalence. The data presented here supplements these findings with the factor of long-term assessment. By tracking the detection of anti-HEV IgM and IgG antibodies over a period of up to 5 years in 370 blood donors, we systematically monitored long-term serology. Surprisingly, a proportion of 7.3% positive anti-HEV IgM (long-term IgM-positive) and 9.1% with negative anti-HEV IgG (seroreversion) were observed five years after index donation. Furthermore, differences were found in the frequency of these phenomena as well as in the quantification of titers between the sexes.The proportion of anti-HEV IgM positive donors was highest shortly after infection with HEV (three months follow-up), and the average IgM S/CO value decreased over the years and fell below the cut-off for a positive value in most donors. These kinetics were to be expected for IgM response [21]. Meanwhile, it should be noted that the serostatus IgM+/IgG− was only rarely detectable, which, however, fits in with an observation already published that anti-HEV IgM and IgG can be detected relatively simultaneously, but have their maximum with a delay [8, 23, 24]. In addition, it was also evident after three months that a large proportion (42.9%) had already seroconverted to IgM−/IgG+ status, which is consistent with a previously published observation in which a median time to disappearance of anti-HEV IgM of 85 days was determined [8].

Nevertheless, contrary to the usual kinetics of disappearing IgM detection after a few weeks, a proportion of 9.1% of anti-HEV IgM-positive donors were found even after five years, if the Euroimmun assay is used as a basis, and 7.3% if the 2:1 rule is applied. Riveiro-Barciela et al. published a similar observation over a period of 36 months in a group of 12 HEV-infected patients with acute hepatitis, 42% of whom still had an anti-HEV IgM-positive result when analyzed with the Mikrogen assay and 17% when using the Wantai assay. In our case, a positive result with the Euroimmun assay was consistent with the Mikrogen assay in 94.7%, while the Wantai assay gave a confirming positive result in 51.6% of cases. Taken together, the Mikrogen assay has a higher detection rate of IgM-positive samples compared to the Wantai assay. Whether this is due to a lower specificity or a higher sensitivity is intensively discussed elsewhere and cannot be conclusively clarified here [25–28]. In addition, our data suggest that the Euroimmun assay sensitivity and specificity is intermediate between these two assays. As the focus of our study was not on calculating the assay sensitivity but on the basic long-term course of the serology, we did not perform deeper comparative studies.

Similar to anti-HEV IgM, the number of positive anti-HEV IgG donations also increases rapidly after the index donation and reaches its maximum after three months. These observations are also consistent with the expected general kinetics of IgG antibodie response and also with descriptions in HEV infected individuals [8, 29]. A decrease in the IgG value is generally to be expected, but on average it did not fall below the detection limits, so that IgG may contribute to immunity against HEV over several years for most donors.

Noteworthy, 36.4% of the donors were IgG-negative five years after their index donation using the Euroimmun assay. Using the 2:1 rule, this only applied to 9.1%. Seroconversion post HEV RNA-positivity was detectable in all returning donors, although in 13 cases this seroconversion was not detectable with the assays provided by Euroimmun but with the other two assays.

Against this background, the Euroimmun assay seem to have a lower detection rate in late-stage post infection than the other two assays, whereby the result of the Mikrogen assay contradicted the result of the Wantai assay more often and yielded a positive result. Particularly in view of the fact that the Euroimmun assay yielded negative results in samples with average lower (semi-) quantitative values in the other two assays, we assume that this assay has a lower sensitivity, which has already been described in a study by Avellon et al. [26]. Contrary to data from other publications, which reported a sensitivity range of 74—97.1% for the Wantai assay and 65–95.3% for the Mikrogen assay, our data suggest that the Mikrogen assay is more sensitive than the Wantai assay [26–28]. In our study, 13 out of 370 donors (3.5%) showed no seroconversion in the Euroimmun assay. In comparison, in a Swiss study, only 1 out of 90 donors (1.1%) who were analyzed with the Wantai assay showed no seroconversion [30]. Therefore, when discussing the results below, a certain inaccuracy of the results depending on the assay used must always be taken into account. Comparative studies have shown that the assessment of the serostatus of HEV-infected persons is variable for different serological assays, which was basis for the decision of our testing strategy [22, 31].

Seroreversion (falling below the cut-off for a positive anti-HEV IgG titre) was observed for a certain proportion of donors, although it is a matter of sensitivity and the interpretation of cut-offs depending on the assay used. Seroreversion rates of 1.9% annually (among HEV seropositive individuals observed over 12 years; no male bias, Mikrogen assay), 15% annually (population-representative HEV serology positive cohort; 19% in females, Wantai assay) or 19% in follow-up samples (HIV- and HEV-coinfected individuals; Wantai assay) have been described in other studies [32–34]. The extent to which an HEV infection once experienced offers lifelong protection or protection against complications in the event of reinfection can only be evaluated through observation over decades demonstrably reinfected people. Whether reinfections occur at all among healthy individuals has not been clarified.

In addition to the question of reactivability at low-level IgG titers, an analysis of the avidity of IgG antibodies, cellular immunity and vaccine-induced immunity is a promising approach in future studies to investigate the immune response to HEV in more detail.

We observed several donations (n = 28) that were anti-HEV IgG positive at the index donation. However, it is not possible to differentiate whether the donors underwent reinfection or were already in a late phase of infection as the serostatus from before the donation was unknown. Nevertheless, it is interesting that in individual cases we were able to determine an IgM−/IgG+ serostatus at the index donation (n = 3) and a subsequent donation showed an IgM+/IgG+ status. The reason for this could be that (a) anti-HEV IgG can be detected before anti-HEV IgM if the titers increase at the same time, but the sensitivities of the assays differ, or that (b) these individuals have been reinfected, particularly since the index donation was already anti-HEV IgG positive.

Another striking feature of our data was the difference in long-term serology between the sexes. This was reflected in both, the qualitative observations and quantitative values. Compared to the general proportion of females in the subcohorts (between 21.3 and 25.9%), a higher proportion of those with long-term IgM (34.2%) and a lower proportion of IgG seroreverts (15.3%) were women. This is interesting against the following background: studies, for example, in German cohorts, have not found significant differences in seroprevalence between men and women, while a higher incidence among men was found when screening for HEV RNA [21, 32]. A study of Bulgarian blood donors shows that although the HEV seroprevalence was similar in men and women, the incidence was twice as high in men in the same cohort [35]. A male bias in immune response is known to occur in various viral infections and is associated with physiological, sex-specific factors such as immunomodulators encoded on the X chromosome or hormone-dependent gene expression regulators [36, 37]. Due to the lack of long-term studies, reference is usually made to the stronger humoral immune response in women shortly after infection. In our data, the long-term differences in antibody formation against HEV were more pronounced. A similar observation was made in the long-term observation of antibodies against SARS-CoV2, with a significantly greater decline in IgG antibody titers in men than in women after one year [38].

In conclusion, the evaluation of seroprevalence and incidence, for example in blood donors, offers interesting conclusions on the epidemiology of HEV. Long-term studies are needed to link these two parameters. Based on the data analyzed in this study with a unique cohort, it should be noted that.

1. IgM positive results without NAT testing do not confirm recent infection (long-term IgM positivity).
2. A negative anti-HEV IgG result does not exclude a prior HEV infection (seroreversion).
3. Sex-dependent antibody response against HEV is contributing to the divergence between sex-based incidence and seroprevalence.

## Data Availability

Data available on request.
